# Selection and mutation on microRNA target sequences during rice evolution

**DOI:** 10.1186/1471-2164-9-454

**Published:** 2008-10-02

**Authors:** Xingyi Guo, Yijie Gui, Yu Wang, Qian-Hao Zhu, Chris Helliwell, Longjiang Fan

**Affiliations:** 1 Institute of Crop Science & Institute of Bioinformatics, Zhejiang University, Hangzhou 310029, PR China; 2 CSIRO Plant Industry, Canberra, ACT 2601, Australia

## Abstract

**Background:**

MicroRNAs (miRNAs) posttranscriptionally down-regulate gene expression by binding target mRNAs. Analysis of the evolution of miRNA binding sites is helpful in understanding the co-evolution between miRNAs and their targets. To understand this process in plants a comparative analysis of miRNA-targeted duplicated gene pairs derived from a well-documented whole genome duplication (WGD) event in combination with a population genetics study of six experimentally validated miRNA binding sites in rice (*O. sativa*) was carried out.

**Results:**

Of the 1,331 pairs of duplicate genes from the WGD, 41 genes (29 pairs) were computationally predicted to be miRNA targets. Sequence substitution analysis indicated that the synonymous substitution rate was significantly lower in the miRNA binding sites than their 5' and 3' flanking regions. Of the 29 duplicated gene pairs, 17 have only one paralog been targeted by a miRNA. This could be due to either gain of a miRNA binding site after the WGD or because one of the duplicated genes has escaped from being a miRNA target after the WGD (loss of miRNA binding site). These possibilities were distinguished by separating miRNAs conserved in both dicots and monocot plants from rice-specific miRNAs and by phylogenetic analysis of miRNA target gene families. The gain/loss rate of miRNA binding sites was estimated to be 3.0 × 10^-9 ^gain/loss per year. Most (70.6%) of the gains/losses were due to nucleotide mutation. By analysis of cultivated (*O. sativa*; *n *= 30) and wild (*O. rufipogon*; *n *= 15) rice populations, no segregating site was observed in six miRNA binding sites whereas 0.12–0.20 SNPs per 21-nt or 1.53–1.80 × 10^-3 ^of the average pairwise nucleotide diversity (π) were found in their flanking regions.

**Conclusion:**

Both molecular evolution and population genetics support the hypothesis that conservation of miRNA binding sites is maintained by purifying selection through elimination of deleterious alleles. Nucleotide mutations play a major role in the gain/loss of miRNA binding sites during evolution.

## Background

MicroRNAs (miRNAs) are endogenously encoded small RNAs that play important roles in regulation of gene expression in animals and plants. The majority of known mature miRNAs are about 20–24 nucleotides long and have been identified in a wide range of eukaryotes, such as fruit fly, nematode, zebrafish, chicken, mouse, human, *Arabidopsis*, maize and rice (reviewed by Bartel [1]). In rice, at least 76 miRNA families consisting of 269 members have been reported (miRBase, Release 11.0, ; [2]) and recently, another 39 new miRNA families have been identified from developing rice grains [3]. Identification of target genes is an essential step in determining the biological functions of miRNAs. Since plant miRNAs recognize their target mRNAs by near-perfect base pairing, computational sequence similarity searches can be used to identify potential targets [4-7].

Selective constraint is defined as the factor by which evolutionary divergence of a functional sequence is reduced due to the action of purifying selection [8]. The basis of the estimation of selective constraint is the comparison of the relative divergence of putatively constrained segments of the genome with that of linked, putatively neutrally evolving sequences. By comparing homologous segments, nucleotide and/or insertion/deletion substitutions are assumed to fall into two classes: neutral, evolving at the same rate as the neutral sequence; or strongly constrained, in which mutations are eliminated unconditionally by natural selection. It is assumed that homologous segments that show significant similarity are under strong selective constraints, while other sequences lacking similarity are evolving free from selective constraints. Based on this assumption, several methods for estimation of selective constraint have been proposed and applied to the coding and non-coding DNAs in invertebrates and mammals (e.g. [9-11]). By comparing recent segmentally duplicated genes we found that strong purifying selection applies to non-coding sequences in rice [12]. Selective constraint has also been detected based on population genetics data from SNP projects in human [9,13,14]. An evolutionary model has been proposed which hypothesises that deleterious SNPs can be observed in nature but most of them will be prevented from reaching a high frequency or going on to be fixed [13]. Thus, regions under purifying selection should have less segregating sites compared with the linked neutral site, which can tolerate deleterious mutations. For example, analysis of the distribution of fitness effects on new mutations in the conserved non-coding sequences in mammals has revealed weak purifying selection [15], whereas in humans, purifying selection is stronger in the conserved miRNA binding sites than in other conserved sequence motifs in the 3' UTRs [13].

An ancient polyploid origin of the rice genome has been well documented [16-20]. The whole genome duplication (WGD) event occurred ~70 million years ago, predating the divergence of cereals (50 million years ago; [21]) from their common ancestor, but postdating the moncot-dicot divergence (~200 million years ago; [22]).

**Figure 1:**
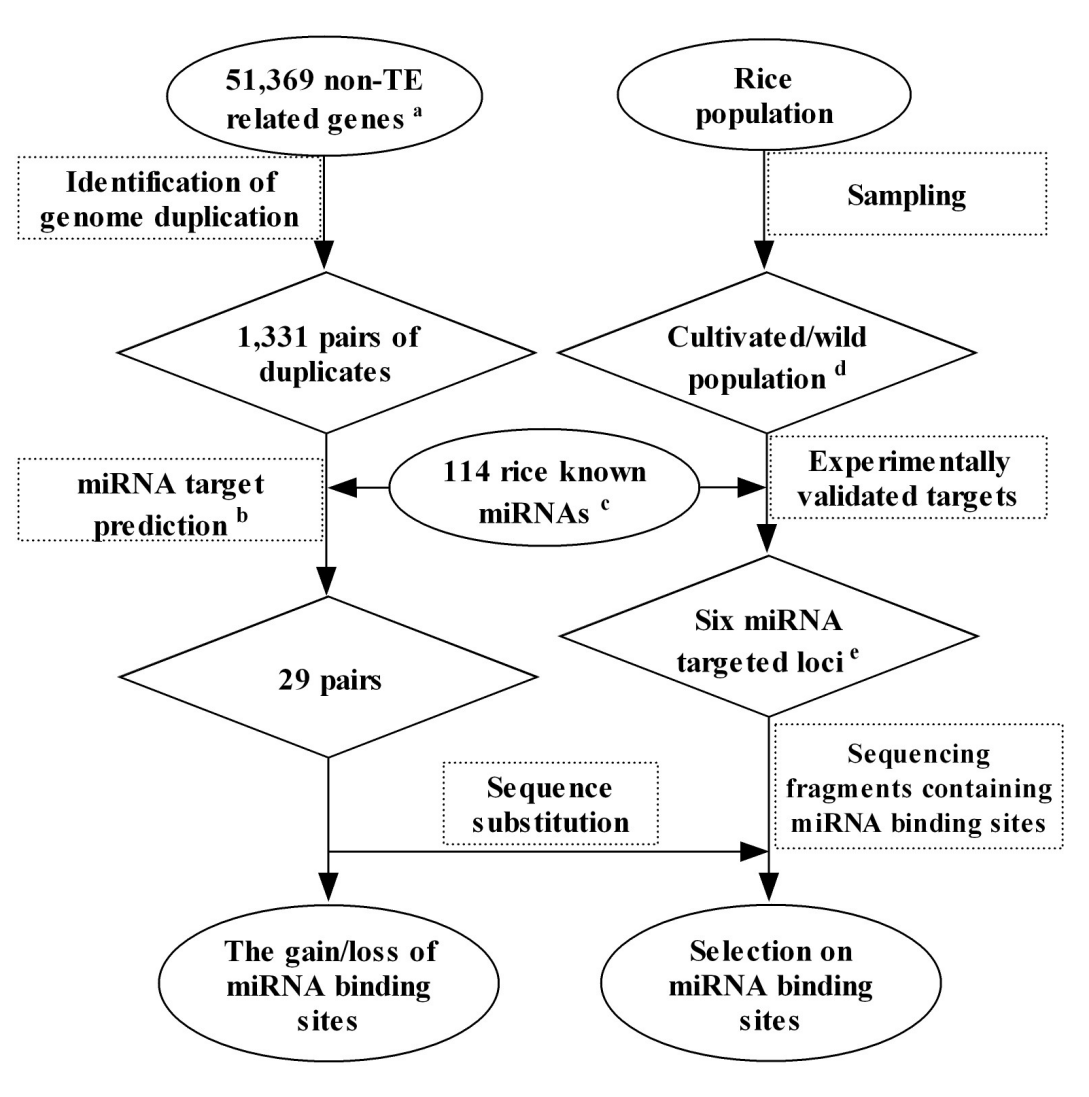
**Flow chart for the methods used in this study**. Two approaches, molecular evolution and population genetics were used to analyze a selection of the miRNA target genes and the gain or loss of miRNA binding sites. a: the rice genome annotation by the TIGR; b: empirical parameters: no mismatch at positions 10 and 11; no more than one mismatch at positions 2–12; no more than two consecutive mismatches downstream of position 13; c: 76 miRNAs from miRBase (Release 11.0, ) and 38 newly identified miRNAs [3]; d: see Additional file 8; e: see Additional file 4.

In this study, the evolutionary pattern of miRNA binding sites in rice was investigated using two different approaches (see Figure 1 for the flow chart): a molecular evolutionary investigation based on WGD paralogs targeted by known rice miRNAs and a population genetics investigation of six experimentally validated miRNA binding sites. Both investigations revealed a highly conserved miRNA binding site and strong evolutionary selection on miRNA binding sites in rice.

## Results and discussion

### Prediction of miRNA targets located on WGD genes

At least nine duplicated blocks from an ancient WGD with retained syntenic duplicate gene copies have been reported [16-20]. A total of 1,331 paralogous gene pairs (Additional file 1) derived from the WGD that occurred ~70 million years ago (mya) was determined following the methods of Guo et al. [12] and Lin et al. [23] from the 51,369 annotated non-transposable element-related rice genes (TIGR Release 5; Figure 1). One hundred and fourteen rice miRNA families, including 76 from miRBase (Release 11.0, ; the predicted miRNAs miR413-420 and miR426 were not included) and 39 newly identified miRNA families [3] were used to predict target genes located on the 1,331 duplicated gene pairs following the method of Rhoades et al. [4] and Schwab et al. [7], which are based on additive, position-dependent mismatch pair penalties. A total of 29 gene pairs from the 1,331 duplicated gene pairs were predicted (see Methods). Of the 29 pairs, 17 have a miRNA binding site on only one of the two duplicated paralogs (Additional file 2) and other 12 have the same miRNA binding site on both duplicated paralogs (Additional file 3). The 29 gene pairs were used for subsequent analysis.

To estimate the accuracy of the predicted miRNA binding sites in the 29 gene pairs or 41 genes, a comparison between the miRNA dataset and a random dataset was carried out using the method developed by Rhoades et al. [4]. For each authentic miRNA, ten cohorts with randomly permuted sequences that had identical sizes and compositions as the authentic miRNAs were constructed. Using the same prediction method for the authentic miRNA, its ten cohorts were searched for complementary sites within the WGD genes using the PatScan program under the same empirical parameters. The result indicated that substantially more complementary sites were found for the authentic miRNA than for its randomized sequences (3.1:48, false positive rate = 0.061). The result suggests that most of the predicted complementary sites should be authentic targets of miRNAs. The predicted miRNA target genes were grouped into functional categories (TIGR GO annotation) and it was found that transcription factors (41%) are the most abundant target genes. These results suggest that the predicted miRNA target genes provide a reliable dataset for the following analysis.

### Selection on microRNA binding sites

Evolutionary changes to miRNA binding sites in rice were investigated through approaches of molecular evolution using the well documented genome duplicated gene pairs targeted by known rice miRNAs and population genetics using a wide range of cultivated and wild rice accessions, respectively (Figure 1).

To detect selection pressure in the regions containing the miRNA binding sites, we divided a miRNA target gene into three parts: the miRNA binding site, its 5' and 3' flanking regions. For each region, the synonymous (*Ks*) and non-synonymous (*Ka*) substitution rates between the two duplicated genes of the 29 gene pairs were estimated. It was found that the *Ks *in the miRNA binding site and its 5' and 3' flanking regions were 0.209, 1.329 and 1.772, respectively (Table 1). A significant reduction in synonymous nucleotide substitute rate was detected in the miRNA binding site compared with its 5' or 3' flanking region (non-parametric Wilcoxon test:

**Table 1:** Estimations of synonymous (*Ks*) and non-synonymous (*Ka*) substitution rates in the miRNA binding sites and their flanking regions

Regions	*Ks*	*p*-value	*Ka*	*p*-value	*Ka/Ks*
5' flanking	1.329 ± 0.256	V = 403, 5.533e-06	0.190 ± 0.036	V = 375, 9.391e-05	0.184 ± 0.035
Binding site	0.209 ± 0.040	/	0.067 ± 0.013	/	0.262 ± 0.133
3' flanking	1.772 ± 0.341	V = 406, 3.995e-06	0.237 ± 0.046	V = 369, 1.639 e-04	0.163 ± 0.031

Non-parameter Wilcoxon test results between the 5' or 3' flanking region and the miRNA binding site were shown.

V = 403, *p*-value = 5.533e-06 and V = 406, *p*-value = 3.995e-06 for the 5' and 3' flanking regions of the miRNA binding site, respectively). A similar result was also observed for the non-synonymous nucleotide substitution rate. These results suggest that the miRNA binding site is more conserved than the regions flanking it; hence there is a stronger selection on the miRNA binding sites than on regions surrounding the miRNA target sites during co-evolution of miRNAs and their target genes. The ratio of *Ka*/*Ks *is usually used to characterize selective evolution of sequences: *Ka*/*Ks *ratios of less than 0.25 indicate purifying selection, values of 1 suggest neutral evolution, and values greater than 1 indicate positive selection [24]. Apparently, purifying selection was observed in rice miRNA target sites and their nearby flanking regions (Table 1). It should be noted that the false positive rate of 9.2% for the predicted miRNA target sites could weaken but not bias our results, because the selection pressure on the falsely predicted miRNA binding sites is expected to be same as that on their flanking regions

Population genetics is a useful tool to detect the evolutionary selection on miRNA target loci [13]. To test whether nucleotide polymorphism frequency in the miRNA binding sites differs significantly from that in the non-binding regions, nucleotide polymorphisms of six experimentally validated miRNA target genes (Additional file 4) were investigated in the cultivated and wild rice populations. Genomic fragments of 650–850-bp containing the binding sites of the miRNAs (miR156::Os08g39890, miR159::Os01g59600, miR390::Os02g10100, miR395::Os03g09930, miR408::Os03g15340 and miR820a::Os03g02010) were amplified and sequenced (Accession nos. EU382760–EU382980). It was found that the SNP density was significantly constrained in the miRNA binding sites compared to their flanking regions in all target genes of the six miRNAs (Table 2). For example, we failed to detect any segregating site in the miRNA binding sites while 9.31 SNPs/kb or 0.20 SNPs/21-nt, and 5.59 SNPs/kb or 0.12 SNPs/21-nt were found at the 5' and 3' flanking regions, respectively. The average nucleotide diversity (π) of the miRNA binding sites (0) is lower than those of their flanking regions (1.53–1.80×10^-3^) in both the cultivated and wild rice populations (Table 2; Additional file 5), suggesting a stronger selection on the miRNA binding sites, a result consistent with that observed in our analysis of duplicated genes. Our results also suggest that the purifying selection is still dominates in cultivated population of rice even although rice has undergone recent strong domestication selection.

**Table 2:** Summary of sequence divergence of six experimentally validated miRNA binding sites in the cultivated rice population. *n*, number of samples; *S*, number of segregating sites; π, average number of pairwise nucleotide differences per site between two sequences [43]; θ, the Watterson estimator of θ per basepair [42].

miRNA	Target gene	*n*	Region	Position	Length (bp)	*S*	π (×10^-3^)	θ (×10^-3^)
miR156	Os08g39890	29	5' flanking	1–197	197	5	5.75	6.46
			Binding site	198–218	21	0	0	0
			3' flanking	219–797	279	6	2.02	2.93

miR159	Os01g59660	29	5' flanking	1–455	455	1	0.15	0.56
			Binding site	456–476	21	0	0	0
			3' flanking	477–778	302	3	2.09	2.54

miR390	Os02g10100	27	5' flanking	1–212	212	2	2.53	2.45
			Binding site	213–233	21	0	0	0
			3' flanking	234–689	456	2	1.04	1.14

miR395	Os03g09930	24	5' flanking	1–36	36	1	2.38	7.65
			Binding site	37–57	21	0	0	0
			3' flanking	58–793	736	4	1.66	1.47

miR408	Os03g15340	25	5' flanking	1–27	27	0	0	0
			Binding site	28–49	22	0	0	0
			3' flanking	50–646	597	1	0.56	0.44

miR820a	Os03g02010	24	5' flanking	1–40	40	0	0	0
			Binding site	41–61	21	0	0	0
			3' flanking	62–610	549	2	1.79	0.98

Average			5' flanking	/	161.2	1.5	1.80	2.85
			Binding site	/	21.2	0	0	0
			3' flanking	/	536.5	3.0	1.53	1.58

Polymorphism data are generally only informative for weak selective effects in recent evolution (such as in the rice lineage), whereas divergence data are potentially informative for stronger selective effects and more distant evolutionary events (e.g. ~70 mya) [13]. From divergence data, we have found the presence of an extensive silent nucleotide reduction within miRNA binding sites compared with their flanking regions. SNP density in the miRNA binding sites was also lower than that in their flanking regions. Both of the results strongly suggest that purifying selection played a major role in maintenance of the conservation of miRNA binding regions to meet the requirement of miRNA-target base pairing for target recognition in the binding regions. The same situation of purifying selection on miRNA binding sites via a population genetics investigation was also reported in natural Arabidopsis population recently [25]. Additionally, conservation of miRNA target sites between distantly related plant species has been suggested in previous studies [26-28].

### The gain and loss of miRNA-target interaction sites

The gain and loss of miRNA-target interaction sites are two important processes during co-evolution of a miRNA and its target(s). The duplicated genes from the WGD provide an opportunity to study the gain/loss dynamics of miRNA binding sites after a duplication event in rice. As mentioned above, of the 29 duplicated gene pairs harbouring miRNA binding sites, 12 pairs have miRNA binding sites on both paralogs, indicating that these genes most likely had been regulated by miRNAs or gained their miRNA binding sites before the WGD and that their miRNA binding sites were maintained after the WGD. For the remaining 17 WGD gene pairs only one paralog of the pair is targeted by a miRNA, indicating a gain or loss of miRNA target or a gain of miRNA itself after the WGD event. For a given miRNA, no matter its interaction with target genes predated divergence of dicots and monocots or after the divergence of dicots and monocots but predating the rice WGD event, if all of its target genes belong to a gene family and some family members do not have miRNA binding sites, this would be evidence for loss of a miRNA binding site after the WGD event, whereas a miRNA binding site on only a single gene of a gene family might indicate an acquisition of miRNA function through gain of miRNA binding site (for the existing miRNAs) or evolving of a new miRNA after the WGD event.

**Figure 2:**
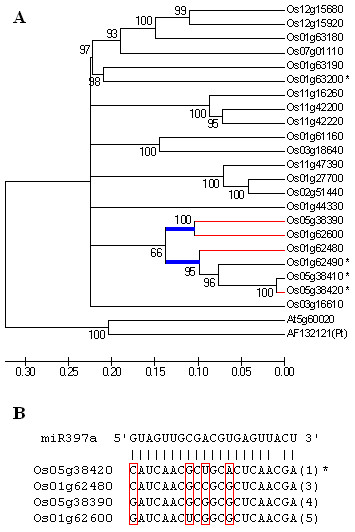
**The loss of miR397 binding sites in a gene family**. **A**: Phylogenetic tree of gene family of L-ascorbate oxidase precursor. The members predicted to be targets of miR397 are labelled with asterisks and the branch node where the WDG event occurred is indicated in blue bold line and the corresponding WGD gene pairs are shown in red lines. Os: *Oryza sativa*; At: *Arabidopsis thaliana *and Pt: *Pinus taeda*; **B**: The alignment of miR397 binding sites of four target genes from the WGD. The numbers of mismatch between miR397 and its binding sites are shown in parentheses. The mutation sites among the four genes are boxed.

All these scenarios were evident in our analysis. miR397 is conserved in dicots and targets L-ascorbate oxidase precursors [29]. Phylogenetic analysis of the target gene family (TIGR ID 3735), with their orthologs from *Arabidopsis *and *pinus *as outgroups, indicated that interactions between miR397 and its targets predated the WGD (Figure 2; Additional file 6). At least four genes of two pairs (Os05g38390/Os01g62600 and Os01g62480/Os05g38420) were derived from the WGD (Additional file 1, 2, 3). There is only one mismatch at position 3 between miR397 and its binding site in Os05g38420 which has been experimentally validated target of miR397 [29], while up to five mismatches are present in the potential miR397 binding site of Os01g62600, which was not a target based on our prediction criteria (Figure 2B). The result suggests that the WGD paralog Os01g62600 has most likely lost its interaction with miR397 or escaped from the control of miR397 after the WGD.

miR156 and miR535 are all conserved in dicots and were predicted to target only one member of their respective target gene families (Additional file 6), suggesting potential gains of binding sites after the WGD. As an example, the putative gain of miR535 binding site was shown in Additional file 7. No sequence similarity was observed between the new target families and the ancestral targets of the conserved miRNAs, indicating they are independent of each other in phylogenetic relationship. This implies gain of a target site via a mechanism not involving inverted repeat derived miRNA/target pair formation [30]. Fahlgren et al. [31] identified recent *MIRNA *loci by comparing similarity between *MIRNA *foldback arms and protein-coding genes. No evidence can be found to support the origin of those conserved miRNAs from their new target families using the method of Fahlgren et al. [31]. Above observations suggest the idea of gain of a target site unrelated to the ancestral ones of the miRNA, a mechanism that is not reported yet. In our data set, all rice-specific miRNAs were predicted to target only one WGD paralog in a single gene family (Additional file 6), which clearly indicate that both miRNAs and their targets arose after the WGD.

The gain or loss rate of the miRNA-target interaction sites after the genome duplication event in rice was estimated. Allowing *p *to be the proportion of gain/loss of the miRNA binding sites between the duplicated genes, we have *p *= 17/(12 × 2 + 17) = 0.415, where 17 is the gain or loss number of the miRNA binding sites, 12 × 2 is the number of conserved miRNA binding sites in two paralogs (Figure 1). Then the expected gain or loss rate of the miRNA binding site can be estimated as the following:

*μ *= *p*/2*t *= 0.415/(2 × 70 × 10^6^) = 3.0 × 10^-9 ^gain/loss per year, where *t *= 70 million years

In rice, Lin et al. [23] estimated that the gain/loss rate of introns is 3.61 × 10^-10 ^per intron per year. A similar gain/loss rate of introns in *Arabidopsis*, 2–3 × 10^-10~12^, has also been reported [32]. Compared with the rate of introns, the gain/loss rate of miRNA binding sites in rice is at least one order higher. It seems to be that relatively long sequences and a complex mechanism might be involved in gene structure evolution. Therefore, it should be more difficult to change a gene structure than a miRNA binding site.

In order to determine the potential mechanisms for the gain/loss of miRNA binding sites, we further investigated and checked the alignments of binding regions in the 17 WGD gene pairs. No gap was found in alignments of 12 (70.6%) gene pairs, suggesting that nucleotide mutation was the main evolutionary force in the gain/loss of miRNA binding sites. Our results indicated that nucleotide mutations together with insertions/deletions are responsible for the gain/loss of miRNA binding sites during co-evolution of miRNAs and their target genes.

## Conclusion

Investigations of both the molecular evolution of WDG gene pairs and population genetics of wild and cultivated rice indicate that pervasive purifying selection might be the major selection constraint for maintenance of the conserved interaction between miRNAs and their binding sites in rice. Our results also revealed that these interactions is a dynamic process because some conserved miRNAs lost their putative target genes derived from the WGD and some conserved miRNAs acquired new target genes, which are usually unrelated to those ancestral targets, after the WGD. The gain/loss rate was estimated to be 3.0 × 10^-9 ^gain/loss per year, with nucleotide mutations playing a major role in the gain/loss of miRNA target sites during evolution.

## Methods

### Plant materials

Forty-five *Oryza *accessions were selected from a wide range of geographical locations to represent a broad range of the genetic diversity within cultivated rice (*O. sativa*) and its wild ancestor, *O. rufipogon*. Detailed information of the 30 domesticated lines (15 *indica *and 15 *japonica *cultivars) and 15 wild lines, which were provided by International Rice Research Institute (IRRI) or China Rice Research Institute, is shown in Additional file 8.

### PCR and DNA sequencing

To investigate sequence variation in the miRNA-targeted protein-coding genes among the selected rice lines, a 650–850 bp genomic fragment that covers the miRNA binding site was amplified from each accession. Primers (Additional file 4) were designed based on the genomic sequence of *japonica *cultivar Nipponbare using Primer3 [33]. The primers were compared to the rice genome sequence to ensure their specificity. Genomic DNA was extracted from fresh rice leaves using a cetyltrimethylammonium bromide (CTAB) protocol [34]. PCR reactions were carried out on a thermocycler (Eppendorf) under the following conditions: 95°C for 5 min, followed by 35 cycles of denaturation at 94°C for 30 s, annealing at 53°C for 30 s and extension at 72°C for 90 s, with a final extension at 72°C for 10 min. PCR products were visualized on 0.8% agarose gel. A product of expected size was amplified from each sample. The amplified products were purified using glassmilk PCR purification kits (BioDev-Tech, China). Purified PCR products were first directly sequenced from both ends using the forward and reverse primer. The PCR products that were failed in direct sequencing were cloned into pGEM-T Easy Vector (TaKaRa) and at least three independent clones were sequenced. Total about 500 PCR products were successfully amplified and sequenced. All sequences have been deposited into GenBank with accession numbers EU382760–EU382980.

### Identification of the genomic duplicated gene pairs

A total of 51,369 non-transposable element-related rice protein sequences (Release 5) were downloaded from the Rice Genome Annotation of TIGR (The Institute of Genomic Research, ). Whole genome duplicated gene pairs were identified using a reciprocal BLASTP [35] search with E-value less than 1e-14 within a distance of 200 kb between collinear gene pairs [12,23]. The dataset containing 285 duplicated gene pairs from chromosomes 11 and 12, which were resulted from a recent duplication about 7 million years ago [36], was excluded. The final dataset contains 1,331 gene pairs (Additional file 1) derived from the WGD.

### Prediction of miRNA binding sites on the duplicated gene pairs

In total, 114 rice miRNA families, 76 (not including miR413-420 and miR426 predicted based on similarities to the predicted *Arabidopsis *miRNAs) from miRBase (Release 11.0, ; [3]) and 39 newly identified miRNA families [3] were used to predict miRNA targets using PatScan [37] on the 1,331 duplicated gene pairs or 2,662 genes following the method of Rhoades et al. [4] and Schwab et al. [7]. G:U and other noncanonical pairs were treated as mismatches. Empirical parameters used in this study: no mismatch at positions 10 and 11; no more than one mismatch at positions 2–12; no more than two consecutive mismatches downstream of position 13. The cut-off vale 4.0 was used. Finally, a set of 29 WGD gene pairs, 17 with miRNA binding sites predicted on only one paralog of the gene pair (Additional file 2) and 12 with miRNA binding sites predicted on both paralogs of the gene pair (Additional file 3) were retained and used for further analysis (Figure 1). As a control, complementary sites were also predicted for 10 cohorts that had identical sizes and base compositions to the miRNAs used but their sequences were randomly permuted.

### Sequence substitution, divergence and phylogenetic analysis

Two protein sequences of each gene pair were aligned using the global sequence alignment program NEEDLE in the EMBOSS package [38]. The alignment result was used to guide the alignment of their corresponding nucleotide sequences, and gaps in the alignment were trimmed. Each gene was then divided into three portions: the 5' flanking region, the miRNA binding site and the 3' flanking region. To detect sequence divergence of each parts in each gene pair, we estimated synonymous (*Ks*) and non-synonymous (*Ka*) substitution rates by using the Yang-Nielson maximum-likelihood method, implemented in the YN00 program of the PAML package [39]. Synonymous sites considered to be of saturation (*Ks *> 5.0) were discarded.

Clustal W [40] was used to align multiple sequences for the construction of phylogenetic trees. Neighbor-joining phylogenies based on the Kimura 2-parameter distance matrix were generated by MEGA version 3.1 [41]. Bootstrap confidence values were obtained by 1000 replicates and were shown. The determination of other family members of miRNA-targeted genes followed the TIGR' annotation . Watterson's estimator of θ [42] and the average pairwise nucleotide diversity π [43] were estimated using DNASP version 4.10.2 [44].

## Authors' contributions

LF and XG conceived and designed the experiments, XG, YG, YW, QZ and LF performed the experiments and analysed the data, CH advised on data analysis, and XG, QZ, CH and LF wrote the paper. All authors have read and approved the final manuscript.

## Supplementary Material

Additional file 1 **The list of 1,331 gene pairs from the whole genome duplication in rice.** Click here for file

Additional file 2 **The 17 WGD gene pairs where miRNA binding sites were predicted on only one paralog of the pair.** Click here for file

Additional file 3 **The 12 WGD gene pairs where miRNA binding site was predicted on both paralogs of the pair.** Click here for file

Additional file 4 **Primers used for amplification of the genomic fragments containing the miRNA binding sites in six experimentally validated miRNA-targeted genes.** Click here for file

Additional file 5 **Summary of the sequence divergence of six experimentally validated miRNA binding sites in the wild rice population.***n*, number of samples; *S*, number of segregating sites; π, average number of pairwise nucleotide differences per site between two sequences [43]; θ, the Watterson estimator of θ per basepair [42]. Click here for file

Additional file 6 **miRNA target predictions in the gene families at Additional file **2. Click here for file

Additional file 7 **The putative gain of miR535 binding site in a gene family.A**: Phylogenetic tree of the gene family (TIGR ID 2995) including Os02g09080. Os02g09080 predicted to be target of miR535 is labelled by an asterisk and the branch node on which the WDG event occurred is indicated in red bold line and the corresponding WGD gene pairs are shown in dot lines. Os: *Oryza sativa *and At: *Arabidopsis thaliana*; **B**: The alignment of the binding sites of gene familiy members. The numbers of mismatch between miRNA and its binding sites are shown in parentheses. Click here for file

Additional file 8 **Accession number and geographic origin of the cultivated and wild rice used in this study.** Click here for file
