# Supplementary material for: Selection and mutation on microRNA target sequences during rice evolution
Source: BMC Genomics. 2008 Oct 2;9:454. doi: 10.1186/1471-2164-9-454 (PMC2567346; doi:10.1186/1471-2164-9-454)
Supplement: Additional file 4 — Primers used for amplification of the genomic fragments containing the miRNA binding sites in six experimentally validated miRNA-targeted genes. [file 1471-2164-9-454-S4.doc]

**Additional file 4** – Primers used for amplification of the genomic fragments containing the miRNA binding sites in six experimentally validated miRNA-targeted genes.

| No. | miRNA | Target gene | Target site | Target protein | Primer ID | Primer sequence | Expected size (bp) | Reference |
| --- | --- | --- | --- | --- | --- | --- | --- | --- |
| 1 | miR156 | Os08g39890 | ORF | SBP domain, transcription factor | mi156-1F | TTCCAAGCAGCGTAAGGAAT | 792 | Xie et al. 2006 |
|  |  |  |  |  | mir156-1R | GGCGCGCATTATTATTCATC |  |  |
| 2 | miR159 | Os01g59660 | ORF | Myb-like DNA-binding domain, transcription factor | mir159F | GATCAGCAGTGCTCCAGTGA | 806 | Luo et al. 2006 |
|  |  |  |  |  | mir159R | AGGAATGGACCAGGATGTTG |  |  |
| 3 | miR390 | Os02g10100 | ORF | Leucine Rich Repeat | mir390F | TTGAGCTTGCAATCGTTGAG | 779 | Sunkar et al. 2005 |
|  |  |  |  |  | mir390R | AGTGGGATTGGGGATTCTTT |  |  |
| 4 | miR395 | Os03g09930 | ORF | ATP sulfurylases | mir395F | CTTGGAGGCACCGATACATT | 843 | Jones-Rhoades and Baretel, 2004 |
|  |  |  |  |  | mir395R | TGGATCAACAGATGGGTCAA |  |  |
| 5 | miR408 | Os03g15340 | ORF | Basic blue protein (Cusacyanin) (Plantacyanin) | mir408F | CTCTCCGAACGAACACACAG | 763 | Sunkar et al. 2005 |
|  |  |  |  |  | mir408R | CACATCACTCATGCCTCACC |  |  |
| 6 | miR820 | Os03g02010 | ORF | cytosine methyltransferase Zmet3 | mir820F | ACAGACGGTGAGGCTGAGAC | 666 | Luo et al. 2006 |
|  |  |  |  |  | mir820R | ACCTCGTCACCAGAACCATC |  |  |

**References**

Jones-Rhoades, M.W. and Bartel, D.P. (2004) Computational identification of plant microRNAs and their targets, including a stress-induced miRNA. Mol Cell 2004, 14:787-799.

Luo, Y.C., Zhou, H., Li, Y., Chen, J.Y., Yang, J.H., Chen, Y.Q. and Qu, L.H. (2006) Rice embryogenic calli express a unique set of microRNAs, suggesting regulatory roles of microRNAs in plant post-embryogenic development. FEBS Lett. 580, 5111-5116.

Sunkar, R., Girke, T., Kumar, P. and Zhu, J-K. (2005) Cloning and characterization of microRNAs from rice. Plant Cell 17, 1397-1411.

Xie, K., Wu, C. and Xiong, L. (2006) Genomic organization, differential expression, and interation of SQUAMOSA promoter-binding-like transcription factors and microRNA156 in rice. Plant Physiol. 142, 280-293.
