# Supplementary material for: Selection and mutation on microRNA target sequences during rice evolution
Source: BMC Genomics. 2008 Oct 2;9:454. doi: 10.1186/1471-2164-9-454 (PMC2567346; doi:10.1186/1471-2164-9-454)
Supplement: Additional file 5 — Summary of the sequence divergence of six experimentally validated miRNA binding sites in the wild rice population.n, number of samples; S, number of segregating sites; π, average number of pairwise nucleotide differences per site between two sequences [43]; θ, the Watterson estimator of θ per basepair [42]. [file 1471-2164-9-454-S5.doc]

**Additional file 5** – Summary of the sequence divergence of six experimentally validated miRNA binding sites in the wild rice population. *n*, number of samples; *S*, number of segregating sites; π, average number of pairwise nucleotide differences per site between two sequences [43]; θ, the Watterson estimator of θ per basepair [42].

| miRNA | Target gene | *n* | Region | Position | Length (bp) | S | π (×10^-3^) | θ (×10^-3^) |
| --- | --- | --- | --- | --- | --- | --- | --- | --- |
|  |  |  | 5' flanking | 1-197 | 197 | 4 | 5.08 | 6.54 |
| miR156 | Os08g39890 | 13 | Binding site | 198-218 | 21 | 0 | 0 | 0 |
|  |  |  | 3' flanking | 219-797 | 579 | 5 | 2.62 | 3.10 |
|  |  |  | 5' flanking | 1-455 | 455 | 3 | 1.02 | 2.13 |
| miR159 | Os01g59660 | 13 | Binding site | 456-476 | 21 | 0 | 0 | 0 |
|  |  |  | 3' flanking | 477-778 | 302 | 3 | 2.09 | 2.54 |
|  |  |  | 5' flanking | 1-212 | 212 | 2 | 3.95 | 3.22 |
| miR390 | Os02g10100 | 11 | Binding site | 213-233 | 21 | 0 | 0 | 0 |
|  |  |  | 3' flanking | 234-689 | 456 | 2 | 1.52 | 1.50 |
|  |  |  | 5' flanking | 1-36 | 36 | 0 | 0 | 0 |
| miR395 | Os03g09930 | 11 | Binding site | 37-57 | 21 | 0 | 0 | 0 |
|  |  |  | 3' flanking | 58-793 | 736 | 3 | 0.75 | 1.41 |
|  |  |  | 5' flanking | 1-27 | 27 | 0 | 0 | 0 |
| miR408 | Os03g15340 | 11 | Binding site | 28-49 | 22 | 0 | 0 | 0 |
|  |  |  | 3' flanking | 50-646 | 597 | 3 | 1.03 | 1.93 |
|  |  |  | 5' flanking | 1-40 | 40 | 0 | 0 | 0 |
| miR820 | Os03g02010 | 11 | Binding site | 41-61 | 21 | 0 | 0 | 0 |
|  |  |  | 3' flanking | 62-610 | 549 | 3 | 2.27 | 1.88 |
|  |  |  | 5' flanking | / | 161.2 | 1.5 | 1.68 | 1.98 |
| Average |  |  | Binding site | / | 21.2 | 0 | 0 | 0 |
|  |  |  | 3' flanking | / | 536.5 | 3.2 | 1.71 | 2.06 |
