# Supplementary figures and images for: Selection and mutation on microRNA target sequences during rice evolution
Source: BMC Genomics. 2008 Oct 2;9:454. doi: 10.1186/1471-2164-9-454 (PMC2567346; doi:10.1186/1471-2164-9-454)

# A

B


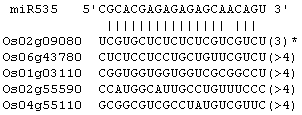


**Additional file 7**

Supplement: Additional file 7 — The putative gain of miR535 binding site in a gene family.A: Phylogenetic tree of the gene family (TIGR ID 2995) including Os02g09080. Os02g09080 predicted to be target of miR535 is labelled by an asterisk and the branch node on which the WDG event occurred is indicated in red bold line and the corresponding WGD gene pairs are shown in dot lines. Os: Oryza sativa and At: Arabidopsis thaliana; B: The alignment of the binding sites of gene familiy members. The numbers of mismatch between miRNA and its binding sites are shown in parentheses. [file 1471-2164-9-454-S7.doc]
