# Supplementary material for: Selection and mutation on microRNA target sequences during rice evolution
Source: BMC Genomics. 2008 Oct 2;9:454. doi: 10.1186/1471-2164-9-454 (PMC2567346; doi:10.1186/1471-2164-9-454)
Supplement: Additional file 8 — Accession number and geographic origin of the cultivated and wild rice used in this study. [file 1471-2164-9-454-S8.doc]

**Additional file 8** – Accession number and geographic origin of the cultivated and wild rice used in this study.

| Number | ID/Accession Number | Cultivar Name | Variety Group# | Country |
| --- | --- | --- | --- | --- |
| 1 | P155 | Kasalath | INDICA | Japan |
| 2 | P370 | Aizhizhan | INDICA | china |
| 3 | P84 | Guangluai4 | INDICA | China |
| 4 | P4 | Minghui63 | INDICA | China |
| 5 | P21 | Teqing | INDICA | China |
| 6 | AAAA02005413 etc* | 93-11 | INDICA | CHINA |
| 7 | IRGC30416 | IR36 | INDICA | PHILIPPINES |
| 8 | IRGC3697 | CO25 | INDICA | INDIA |
| 9 | IRGC9175 | JC93 | INDICA | INDIA |
| 10 | IRGC7755 | KALUKANTHA | INDICA | SRI LANKA |
| 11 | IRGC8231 | GIE57 | INDICA | VIETNAM |
| 12 | IRGC26872 | BINULAWAN | INDICA | PHILIPPINES |
| 13 | IRGC27748 | KHAO DAWK MALI 105 | INDICA | THAILAND |
| 14 | IRGC43400 | ILIS AIR | INDICA | INDONESIA |
| 15 | IRGC58930 | CHHOTE DHAN | INDICA | NEPAL |
| 16 | IRGC 55471 | Chodongji | TEMPERATE JAPONICA | South Korea |
| 17 | IRGC 27630 | Darmali | TEMPERATE JAPONICA | Nepal |
| 18 | IRGC 8264 | Hu Lo Tao | TEMPERATE JAPONICA | China |
| 19 | IRGC 27716 | Kaw Luyoeng | TEMPERATE JAPONICA | Thailand |
| 20 | IRGC 40748 | Nep Hoa Vang | TEMPERATE JAPONICA | Vietnam |
| 21 | IRGC 38690 | NPE 253 | TEMPERATE JAPONICA | Pakistan |
| 22 | IRGC 1107 | Ta Hung Ku | TEMPERATE JAPONICA | China |
| 23 | IRGC 33888 | Yelaik Meedon | TEMPERATE JAPONICA | Burma |
| 24 | Os02g10100 etc* | Nipponbare | TEMPERATE JAPONICA | Japan |
| 25 | 14455 | Balila | TEMPERATE JAPONICA | Italia |
| 26 | IRGC 27869 | Chahora 144 | TROPICAL JAPONICA | Pakistan |
| 27 | IRGC 43372 | CICIH BETON | TROPICAL JAPONICA | Indonesia |
| 28 | IRGC 24225 | KHAO HAWM | TROPICAL JAPONICA | Thailand |
| 29 | IRGC 2545 | KOTOBUKI MOCHI | TROPICAL JAPONICA | Japan |
| 30 | IRGC 6264 | N22 | TROPICAL JAPONICA | India |
| 31 | IRGC 81886 |  | O. rufipogon | India |
| 32 | IRGC 82988 |  | O. rufipogon | China |
| 33 | IRGC 83818 |  | O. rufipogon | Vietnam |
| 34 | IRGC 89019 |  | O. rufipogon | Cambodia |
| 35 | IRGC 93044 |  | O. rufipogon | Cambodia |
| 36 | IRGC 93208 |  | O. rufipogon | Nepal |
| 37 | IRGC 100588 |  | O. rufipogon | Taiwan |
| 38 | IRGC 103423 |  | O. rufipogon | Sri lanKa |
| 39 | IRGC 104308 |  | O. rufipogon | Myanmar |
| 40 | IRGC 104624 |  | O. rufipogon | China |
| 41 | IRGC 104730 |  | O. rufipogon | Thailand |
| 42 | IRGC 104802 |  | O. rufipogon | Thailand |
| 43 | IRGC 105656 |  | O. rufipogon | India |
| 44 | IRGC 105696 |  | O. rufipogon | Nepal |
| 45 | IRGC 106168 |  | O. rufipogon | Vietnam |

* GenBank and TIGR accession numbers for the sequences containing the corresponding miRNA-targeted genes from the Nipponbare and 9311 genome sequencing projects. Also see Additional File 5.

# Classification according to Garris *et al*. (2005, *Genetics*)
